# Depressive symptoms among immigrant and Canadian born mothers of preterm infants at neonatal intensive care discharge: a cross sectional study

**DOI:** 10.1186/1471-2393-13-S1-S11

**Published:** 2013-01-31

**Authors:** Marilyn Ballantyne, Karen M Benzies, Barry Trute

**Affiliations:** 1 School of Nursing, Faculty of Health Sciences, McMaster University, Hamilton, ON, Canada L8S 4K1; 2 Faculty of Nursing, University of Calgary, Calgary, AB, T2N 1N4, Canada; 3 Faculty of Social Work, University of Calgary, Calgary, AB, T2N 1N4, Canada

## Abstract

**Background:**

Mothers of preterm infants are considered at higher risk for depressive symptoms, higher than for mothers of healthy term infants. Predictors of depressive symptoms in mothers of preterm infants are not yet well established. Immigrant mothers of term infants have higher prevalence of depressive symptoms than Canadian born mothers but the relative prevalence for immigrant mothers of preterm infants is unknown. This study had two aims: (i) to investigate the prevalence of depressive symptoms in immigrant as compared to Canadian born mothers of preterm infants, and (ii) to determine what factors are associated with depressive symptoms in mothers of preterm infants.

**Methods:**

This is a multi-site, cross sectional study of mothers whose preterm infants required hospitalization in neonatal intensive care unit (NICU). Consecutive eligible mothers (*N* = 291) were recruited during the week prior to their infant’s NICU discharge. Mothers completed a self-administered questionnaire booklet of validated psychosocial/cultural measures including the Center for Epidemiological Studies Depression Scale (CES-D), Parental Stressor Scale:NICU, General Functioning Subscale of the McMaster Family Assessment Device, Social Support Index, and Vancouver Index of Acculturation; and demographic characteristics questions. Infant characteristics included gestational age, birth weight, sex, singleton/multiple birth, and Score for Neonatal Acute Physiology-II.

**Results:**

Immigrant mothers (*N* = 107), when compared to Canadian born mothers (*N* = 184), reported more depressive symptoms, poorer family functioning, less social support, and less mainstream acculturation. Hierarchical regression for a subsample of 271 mothers indicated that single parent status, high stress, poorer family functioning, and less social support were associated with increased depressive symptoms and accounted for 39% of the variance on the CES-D. Immigrant status did not contribute significantly to the final regression model.

**Conclusions:**

Immigrant mothers of preterm infants are at increased risk for depressive symptoms. For immigrant and Canadian born mothers of preterm infants hospitalized in NICU and particularly for single mothers, interventions to reduce stress and increase family functioning and social support may reduce depressive symptoms. Given the effects of depression on maternal health and functioning, such an intervention may improve child outcomes.

## Background

Postnatal depression affects 7 - 19% of mothers of healthy term infants [1,2] within the first year after childbirth [1]. Mothers of preterm infants (< 37 weeks gestation) are at greater risk for depression than mothers of term infants [3]. Between 28 and 66% of mothers of preterm infants reported depressive symptoms [4-9] severe enough to meet the criteria for a diagnosis of depression (i.e., above the cutoff for the specific depression measure) [1,10]. Mothers of very preterm (< 30 weeks), medically fragile infants had sustained symptoms of depression for up to 2 years [11,12].

Maternal depression is of concern not only because of its effects on the mother’s health and functioning, but also because it is associated with negative child developmental outcomes including cognitive delay, insecure attachment, and difficulties with behavioural and emotional development [1]. Preterm infants are particularly vulnerable to the effects of maternal depression in addition to an increased risk for developmental delays due to their immaturity, medical illness, and biological risk [13].

There is a significant gap in the current literature regarding the risk factors for depression in immigrant mothers [1,14]. This is an important question as many countries have a significant proportion of immigrants. In Canada, one in five individuals are identified as immigrants [15]. Immigrant women are at increased risk for preterm birth [16,17], a risk more strongly associated with higher education status (e.g., university education) [17]. The risk for immigrant women increases with increased time spent in Canada, especially for those who lived in poor neighbourhoods [16]. Recent Canadian research indicated that immigrant women were also at increased risk for depressive symptoms [14,18-22] and low received social support [22]. Measurement of immigration status varied greatly across studies, and typically was based on language spoken in the home, country of origin and/or length of residence in Canada. Acculturation or adaptation to the cultural beliefs and values of the new country were not considered. In Canada, where cultural pluralism is promoted, it is essential to address bi-dimensional acculturation: degrees of identity within mainstream and heritage cultures [23]. Acculturation is viewed as a more valid and predictive construct than measures of ethnicity, language, time in country, or race [24]. To date, no study has addressed immigration and/or acculturation factors and their association with depressive symptoms in mothers of preterm infants. The relative prevalence of depressive symptoms for immigrant mothers of preterm infants is also unknown.

In prior meta-analyses, the strongest predictors of depression for mothers of term infants included stressful life events [10,25,26], a poor marital relationship [10,26], lack of social support [10,25,26], previous history of depression [10,25,26], and anxiety or depression during pregnancy [10,25,26]. The maternal factors associated with depression in mothers of preterm infants are considered to be similar to those found in the literature for mothers of term infants [3]. Yet, maternal factors such as lack of social support, family functioning, and poor marital relationship have not been adequately studied in mothers of preterm infants.

Few infant-specific factors were considered as reported in meta-analyses [1,10,25,26]. Only difficult infant temperament and childcare stress [26] or obstetric factors (e.g., premature delivery) [25] were addressed. These infant factors were less strongly associated with depressive symptoms in mothers of term infants [25,26]. Mothers of preterm infants experience additional stress related to their preterm infant’s illness and early gestational age (GA). Evidence for the contribution of the infant’s severity of illness to maternal depression was conflicting and inconclusive [3] with reports of both association [6,11,27] and non-association [8,9,28-30] with maternal depressive symptoms. The findings of these studies are limited by the use of non-validated clinical illness measures [9] or infant demographic characteristics and/or clinical diagnoses as a proxy for illness severity [6,8,11,27-29]. Given this conflicting evidence, further research is required using validated measures to determine if the infant’s severity of illness is a risk factor that contributes to maternal depressive symptoms.

The two aims of the current study were: (i) to investigate the prevalence of depressive symptoms in immigrant as compared to Canadian born mothers of preterm infants, and (ii) to determine what factors are associated with depressive symptoms in mothers of preterm infants hospitalized in the neonatal intensive care unit (NICU). The factors included demographic characteristics (e.g., maternal and infant), psychosocial context factors (i.e., stress, family functioning, and social support); and factors not adequately or previously studied (i.e., immigrant status, bi-dimensional acculturation and severity of infant illness).

## Methods

### Sample

The current study employed data gathered in a larger study of mothers whose infants required NICU hospitalization [31]. Study participants were recruited between November 2006 and July 2008 from three Canadian tertiary-level NICUs located in Toronto and Hamilton. These cities are located in the area of the province of Ontario where 80% of all provincial births occur. Ontario is the most ethnically diverse Canadian province with 42 - 45% with of all immigrants residing there [32]. Mothers had universal access to care through the publically funded Canadian health insurance system. Criteria for enrollment in the larger study were all mothers who assumed primary responsibility for their infant and whose infant required NICU admission (preterm [<37 weeks] and term). Of the 679 eligible mothers, 87 mothers were excluded (53 [7.8%] were non-English speaking; and 34 [5%] were too ill [e.g., critically ill and hospitalized] and/or denied custody of their infant). An additional 76 mothers (11%) were not invited to participate because of unavailability of the mother or research team. In all, 550 mothers were approached to participate of which 363 participated (66%) in the larger study. For this study, a subset of mothers whose infants were born preterm (*N* = 291) were included.

### Procedures

Research ethics committees of three participating hospitals and the affiliated university approved the study. Consecutive eligible mothers were recruited through the NICUs during the week prior to their infant’s planned discharge. This is a time point that represents the infant’s transition to illness recovery and allows comparisons with previous studies, the majority of which were conducted at or near the point of NICU discharge. After the research nurse conducted written informed consent, mothers completed a self-administered questionnaire booklet at home or in a private location in the NICU. Mothers placed the completed questionnaires in a sealed envelope at the infant’s bedside. The research nurse circulated daily to address questions and collect questionnaires. Study participants completed the maternal questionnaire with < 3% missing data. All mothers had ready access to professional mental health support and counseling (e.g., social workers and psychiatrists) in their respective NICU. Infant characteristics including GA, birth weight (BW), sex, single/multiple birth, and severity of illness (SNAP-II) were obtained from the NICU health record by the research nurse.

### Measurement

The booklet addressed maternal demographic characteristics including age, marital status, parenting status, education, employment, income adequacy (i.e., annual household income that takes the number of person living in the home into account), language in the home, and country of birth, along with five previously validated psychosocial/cultural measures. All measures were reviewed for content validity and feasibility by eight experts. They were pilot tested with 19 mothers (9 immigrant, 10 Canadian-born) who met the eligibility criteria to ensure acceptability and feasibility (e.g., simplicity of language, ease of reading, and time to complete). These 19 mothers were included in the overall study since no changes were made to the measures or procedures.

#### Center for Epidemiologic Studies Depression Scale (CES-D)

The CES-D [33] is a 20-item self-report scale of depressive symptoms with ratings on a 4-point Likert scale that ranges from “rarely or none of the time (less than 1 day in the past week)” to “most of the time (5 – 7 days)” related to the frequency of feelings or behaviours such as the blues, loneliness, and sadness; not feeling as good as others or thinking one’s life is a failure; difficulty concentrating; and sleep and appetite problems. The CES-D was originally validated in the general adult population [33]. It has been used extensively in studies of parents of premature and ill infants [4-6,34-39], allowing comparisons of our study results to other research. The CES-D is not diagnostic for depression but provides an estimate of depressive symptoms. Scores range from 0 to 60. A score of 16 or greater indicates high risk for depression [40,41]. Cronbach’s alphas of .87 to .91 were reported in mothers of preterm infants [6] and .88 to .90 in mothers of medically fragile infants [37]. For this study, Cronbach’s alpha was .90.

#### Parental Stressor Scale: NICU (PSS:NICU)

The PSS:NICU [42] is a validated 34-item scale that measures parents’ stress during their infant’s NICU hospitalization related to alterations in parental role, the infant’s appearance and behaviour, and the NICU environment. Mothers rated their overall experience on a 5-point Likert scale, from 1 (*not at all stressful*) to 5 (*extremely stressful*). Scale scores are calculated by averaging stress responses for the items on each scale and for the total scale to determine the overall stress level. Higher scores indicate higher stress. Stress scores were positively correlated with State Anxiety (r = 0.45, p < .001) [42]. High internal consistency scores were reported for the total scale at .89 (Cronbach’s alpha) [6,42]. For this study, Cronbach’s alpha was .88.

#### General Functioning Subscale of the McMaster Family Assessment Device (FAD-GF)

The FAD-GF [43] is a 12-item scale that measures the family’s ability to make the adjustments required in the family system as a result of the stressor event (e.g., birth of a preterm infant) to maintain balance and family function [44]. Higher scores signify more family dysfunction (> 26 indicates low ability or inability to adjust and function [i.e., pathology]) [44]. Items are rated on a 4-point Likert scale with anchors ranging from 1 (*strongly disagree*) to 4 (*strongly agree*). Related to reliability, internal consistency was .86 (Cronbach’s alpha) and a split-half coefficient (Guttman) was .83 based on a sample of 1,822 families in the Ontario Child Health Study [43,44]. Cronbach’s alpha for mothers of high-risk infants ranged from .81 to .84 [5,45,46]. For this study, Cronbach’s alpha was .87.

#### Social Support Index (SSI)

The SSI [47] is a 17-item scale measuring the degree to which families perceive their family and community as a source of support [47]. Community could mean, for example, their neighbourhood, network of friends, or religious groups. Using a 5-point Likert scale, respondents indicate the degree to which they agree or disagree with statements about their support resources and as a provider of emotional and esteem support. For example, “If I had an emergency, people I know in this community would be willing to help” and “The things I do for members of my family and they do for me make me feel part of this very important group.” Cronbach’s alpha was .82 and test-retest reliability was .83. For this study, Cronbach’s alpha was .86. The SSI is correlated with family well-being and sense of fit within the community (*r* = .40) [47].

#### Vancouver Index of Acculturation (VIA)

The VIA [23] is a bi-dimensional, 20-item, self-report scale used to assess participation in or identification with mainstream (dominant) and heritage (non-dominant) cultures. Each subscale has 10 items with identical wording except for the culture. For example, “I often participate in my heritage cultural traditions” and “I often participate in mainstream North American cultural traditions.” The items assess three domains: values, social relationships, and adherence to traditions. The VIA items are rated on a 9-point Likert scale with anchors ranging from 1 (*strongly disagree*) to 9 (*strongly agree*). Higher scores indicate greater acculturation (maximum = 9). Cronbach’s alphas ranged from .85 to .89 for the mainstream subscale and .91 to .92 for the heritage subscale for a diverse sample of acculturating adults (*N* = 728; e.g., East, South and South East Asian, Italian, and Arabic) [23]. Individuals who identified more strongly with mainstream culture had better psychosocial adjustment (e.g., less depression) than those who identified with heritage culture [23]. For this study, Cronbach’s alphas were .89 for both subscales.

#### Score for Neonatal Acute Physiology-II (SNAP-II)

The SNAP-II is a composite measure of newborn illness severity measured at NICU admission [48-50]. It is a physiology-based score that uses 34 routinely available vital signs and laboratory test results. The SNAP-II was developed and validated prospectively on 25,429 admissions in 30 Canadian NICUs. SNAP-II correlates with therapeutic intensity (*r* = .78) and length of stay (*r* = .59) [49].

### Data analysis

First, comparisons were made between immigrant and Canadian born mothers on the psychosocial/acculturation measures using Student *t* tests. Bivariate Pearson’s correlation and hierarchical linear regression were used to examine the relationships between the dependent (CES-D scores) and independent variables. Significance level of *p* < .05 was set for all statistical tests. Linearity and collinearity were evaluated prior to multiple regression analysis. Analyses were conducted with Statistical Package for Social Sciences (SPSS) – Version 20. Mothers of multiple births were included once with their lesser BW infant.

## Results

Characteristics of immigrant and Canadian mothers (*N* = 291) and their infants are shown in Table 1. Overall, the majority of mothers lived in two-parent families, and were graduates of college or university, employed prior to their infant’s birth, spoke English at home, and had singleton infants. Immigrant mothers were older, had lower income adequacy, and spoke proportionately less English at home than Canadian born mothers.

**Table 1:** Characteristics of study population based on immigrant versus Canadian born status

	Immigrant *N* = 107	Canadian Born *N* = 184
Variable	Mean (*SD*)	Mean (*SD*)
**Maternal Characteristics**		
Mean maternal age, years ^a^	33.28 (5.76)	31.64 (5.92)
	Frequency (%)	Frequency (%)
Single parent	10 (9.4)	13 (7.1)
First time parent	63 (59.4)	114 (62.0)
Employed at time of delivery	91 (85.0)	157 (85.8)
Education ^b^		
No high school diploma	7 (6.7)	13 (7.1)
High school graduate or some college	31 (29.5)	54 (29.3)
College or university graduate	67 (63.8)	117 (63.6)
Income adequacy ^c^		
Low	5 (4.9)	5 (2.8)
Lower middle	9 (8.8)	18 (9.9)
Middle	31 (30.4)	24 (13.3)
Upper middle	24 (23.5)	50 (27.6)
High	33 (32.4)	84 (46.4)
English as 1^st^ language in the home	60 (56.1)	180 (97.8)
		
**Infant Characteristics**	Mean (*SD*)	Mean (*SD*)
Mean gestational age, weeks	27.91 (3.22)	28.62 (3.32)
Mean birth weight, grams	1034.22 (472.74)	1194.92 (641.58)
Median SNAP-II score (IQR)	9 (17)	7 (14)
		
Gestational age by preterm categories	Frequency (%)	Frequency (%)
Extremely preterm, < 28 weeks GA	61 (57.0)	75 (40.8)
Very preterm, 28 to <32 weeks GA	31 (29.0)	72 (39.1)
Moderate/late preterm, 32 to < 36 weeks GA	15 (14.0)	37 (20.1)
Male	52 (48.6)	86 (46.7)
Multiples (e.g., twins or triplets)	12 (11.2)	27 (14.7)

*Note:* Income adequacy based on annual household income and number of persons in the home

SNAP-II = Score for Neonatal Acute Physiology-II; IQR = Interquartile range

^a^ Four missing data (2 immigrant, 2 Canadian born)

^b^ Two missing data (2 immigrant, 0 Canadian born)

^c^ Eight missing data (5 immigrant, 3 Canadian born)

Immigrant mothers were ethnically diverse. Seventy percent of immigrant mothers were from the Caribbean (e.g., Jamaica, Barbados, Trinidad, St Lucia), South Asia (e.g., India, Pakistan, Bangladesh, Sri Lanka), Southeast Asia (e.g., Philippines, Vietnam), Western Europe (e.g., United Kingdom, Portugal) and Africa (e.g., Ghana, Nigeria, Egypt) comparable with reported immigration patterns to Canada during the study period [15].

### Psychosocial and acculturation measures – comparison between groups (Aim 1)

The mean CES-D score for immigrant mothers was significantly higher than for Canadian born mothers (*t* = 3.02, *p* = .002) (see Table 2). For example, the mean CES-D score for immigrant mothers was 20.7 as compared to 16.7 for Canadian born mothers. Fifty-nine percent of immigrant and forty-seven percent of Canadian born mothers had scores indicative of high risk for clinical depression (> 16). The Cronbach’s alpha for the CES-D was consistent with other studies of mothers of ill and preterm infants. Immigrant mothers had FAD-GF scores indicative of significantly poorer family functioning than Canadian born mothers (*t* = 2.50, *p* = .01) and significantly lower SSI scores (*t* = -4.25, *p* < .001), indicative of lower social support. Mean scores on the FAD-GF were well below the clinical cut-off (>26). Immigrant mothers reported significantly lower mainstream cultural identity on the VIA (*t* = -5.19, *p* < .001) than Canadian born mothers with no differences based on heritage cultural identity (*t* = 1.15, *p* = .25). Immigrant mothers’ scores on the mainstream and heritage subscales were quite similar. The mean stress scores on the PSS:NICU were similar (*t* = 1.36, *p* = .17) for immigrant and Canadian born mothers.

**Table 2:** Scores on psychosocial measures based on immigrant versus Canadian born status

	Immigrant *N* = 107	Canadian Born *N* = 184		
Variable	Mean (*SD*)	Mean (*SD*)	*t* score	*p*-value

Depression (CES-D) ^a^	20.73 (11.41)	16.65 (10.08)	3.02	.002
Stress (PSS:NICU) ^a^	3.22 (0.81)	3.09 (0.74)	1.36	.17
Family Functioning (FAD-GF) ^b^	19.36 (4.96)	17.80 (5.19)	2.50	.01
Social Support (SSI) ^c^	47.65 (9.47)	52.19 (8.26)	-4.25	< .001
Acculturation (VIA-Mainstream) ^d^	6.39 (1.18)	7.12 (1.12)	-5.19	< .001
Acculturation (VIA-Heritage) ^a^	6.63 (1.31)	6.43 (1.46)	1.15	.25

*Note*: t score = independent samples *t*-test; CES-D = Center for Epidemiologic Studies Depression Scale; PSS:NICU = Parental Stressor Scale: neonatal intensive care unit; FAD-GF = Family Assessment Device – General Functioning Scale; SSI = Social Support Index; VIA = Vancouver Index of Acculturation – Mainstream and Heritage Scales.

^a^ Seven missing data (CES-D, 4 immigrant, 3 Canadian born; PSS:NICU, 2 immigrant, 5 Canadian born; VIA-Heritage, 2 immigrant, 5 Canadian born)

^b^ Two missing data (2 immigrant, 0 Canadian born)

^c^ Four missing data (2 immigrant, 2 Canadian born)

^d^ Six missing data (2 immigrant, 4 Canadian born)

### Pearson’s correlations (Aim 2)

Stress, family functioning, social support, mainstream acculturation identity, immigrant status, and single parent status were significantly correlated with depressive symptoms on the CES-D (see Table 3). CES-D scores increased with increased stress, poorer family functioning, low social support, less mainstream acculturation identity, immigrant versus Canadian born status, and single versus married status. Maternal variables including age, first time parent, employment, education, income adequacy, and English as first language in the home, and infant variables (i.e., GA, BW, sex, singleton/multiples, and severity of illness [SNAP-II score] were not correlated with depressive symptoms.

**Table 3:** Summary of Pearson’s correlations and descriptive statistics for validated measures

Variable	1	2	3	4	5	6	7	8
1. CES-D	-							
2. PSS:NICU	.48**	-						
3. FAD-GF	.35**	.16**	-					
4. SSI	-.44**	-.15*	-.48**	-				
5. VIA-Mainstream	-.15*	-.02	-.16**	.26**	-			
6. VIA-Heritage	.00	.04	-.08	.11	.35**	-		
7. Immigrant/Canadian born	-.18**	-.08	-.15*	.24**	.30**	-.07	-	
8. Single parent	.18**	.07	.21**	-.16**	-.12	-.10	-.04	-
								
M	18.13	3.1	18.37	50.53	6.85	6.50		
*SD*	10.74	.77	5.15	8.98	1.19	1.40		
Range	0-49	1-4.74	10-42	16-68	4-9	1-9		

*Note*: CES-D = Center for Epidemiologic Studies Depression Scale; PSS:NICU = Parental Stressor Scale: Neonatal Intensive Care Unit; FAD-GF = Family Assessment Device – General Functioning Scale; SSI = Social Support Index; VIA = Vancouver Index of Acculturation - Mainstream and Heritage Scales; M = Mean, *SD* = Standard deviation

*p < .05, **p < .01, *** p < .001

### Multiple linear regression (Aim 2)

Hierarchical linear regression was employed to test the variance explained in depressive symptoms as measured by the CES-D for the 271 mothers with complete data for all variables (Table 4). Variables with a significant zero-order correlation with the CES-D were tested as potential predictors of mothers’ symptoms of depression in the hierarchical regression analysis. Since immigrant status and acculturation were correlated (*r* = .30, *p* < .001), immigrant status was used to represent this construct as its correlation with depressive symptoms was higher. Variables were entered in blocks: Step 1, single parent status and immigrant/Canadian born status (maternal characteristics); Step 2, PSS:NICU (maternal internal psychosocial context); and Step 3, FAD-GF and SSI [1] (maternal external psychosocial context).

**Table 4:** Hierarchical multiple regression analysis predicting maternal depressive symptoms (*N*= 271)

Variable		*B*	*SE B*	β
				

Step 1				
	Single parent	8.25	2.39	.20**
	Immigrant/Canadian born	- 4.09	1.30	- .18**
				
Step 2				
	Single parent	7.38	2.12	.18**
	Immigrant/Canadian born	- 3.03	1.16	- .14*
	Stress (PSS:NICU)	6.42	.74	.45***
				
Step 3				
	Single parent	4.63	1.98	.11*
	Immigrant/Canadian born	- 1.31	1.09	- .06
	Stress (PSS:NICU)	5.62	.68	.40***
	Family functioning (FAD-GF)	.28	.11	.14*
	Social support (SSI)	- .34	.07	- .28***
				

*R*^2^ = .07 for Step1, Δ*R*^2^ = .20 for Step 2, Δ*R*^2^ = .12 for Step 3

*p < .05, **p < .01, *** p < .001

In Step 1, single parent and immigrant status contributed significantly to the model and explained 7% of the total variance in CES-D scores. In Step 2, PSS:NICU contributed significantly and added an additional 20% to the total variance explained. In Step 3, FAD-GF and SSI contributed significantly and added an additional 12% to the variance explained. Together, four variables (single parent, PSS:NICU, FAD-GF, and SSI) explained 39% of the total variance in CES-D scores. Thus, the final model indicated that being a single parent and having higher stress, poorer family functioning, and lower social support, but not immigrant status, were significant predictors of maternal depressive symptoms.

### Discussion

Findings from this study contribute to our knowledge about the impact of preterm birth and NICU hospitalization on the mother’s risk for depressive symptoms with particular attention to the impact of immigrant status. Our findings indicate that 59% of immigrant and 47% of Canadian born mothers in this sample report depressive symptoms above the cutoff. These proportions are similar to studies of mothers of preterm infants with comparable GA (28 weeks mean GA) where 40 to 63% of mothers had mean depression scores above the cutoff for the measure used and indicating high risk of clinical depression [6,8,9,28]. Only two studies reported either low risk for depressive symptoms [51] or no difference in risk for mothers of preterms as compared to term controls [27] during NICU hospitalization. The findings of these two studies may have differed because the mothers had preterm infants of higher GA (e.g., 31 to 35 weeks mean GA) who typically have lower illness severity than younger GA infants [49].

To our knowledge, this is the first study to address immigrant mothers with preterm infants and their psychosocial adjustment. These mothers had significantly increased depressive symptoms as compared to Canadian born mothers. Because mothers were recruited from consecutive eligible infants, the sample is more likely to represent the population of women in Ontario who have children born preterm, although the participation rate was only 66%. The proportion (37%) of our sample who were immigrant mothers is similar to the proportion of Canadian immigrants (42%) who reside in Ontario [32] and their origins are similar to the Canadian immigration pattern. The findings related to depressive symptoms of immigrant mothers of preterm infants are similar to other Canadian studies of immigrant women with healthy term infants who were at greater risk for depressive symptoms than Canadian born mothers [14,18-20,52]. However, more research is warranted to guide our understanding of how cultural identity within immigrant mothers and the birth of a preterm infant uniquely and collectively contribute to maternal depressive symptoms in order to inform and provide effective interventions. Women who could not read English were excluded from the study, so this population is not represented in the findings.

The second aim of the study was to identify factors associated with depressive symptoms in mothers of preterm infants. Three psychosocial contextual factors (stress, family functioning, and social support), and one maternal characteristic (single parent status) significantly predicted maternal depressive symptoms. Maternal perception of stress was one of the most significant psychosocial predictors of depressive symptoms for all mothers of preterm infants in this large cross sectional study. This finding is consistent with other studies where stress was associated with depressive symptoms in mothers with preterm infants during NICU hospitalization [6,8,9,28]. Only Miles and colleagues [9] reported an association between stress and depression related to the mother’s worry. While we did not address mother’s worry, stress in our study was related to the alterations in the parental role, appearance and behavior of their infant, and the sights and sounds of the NICU. Other concurrent stressors such as additional changes within the family, job loss, and family health concerns were not addressed in this study. Mean stress scores were similar between immigrant and Canadian born mothers indicative that having a preterm infant hospitalized in the NICU was stressful for all parents regardless of their immigrant status.

Unique to this study was the finding that low family functioning was associated with depressive symptoms in mothers of preterm infants, consistent with studies in mothers of term infants [1,3,10,25,26]. There are no studies of mothers of preterm infants that adequately addressed family functioning (i.e., the family’s ability to adjust and function to the stressful event of preterm birth and NICU hospitalization). Instead, Miles and colleagues [9] investigated maternal perception of father’s involvement in care, a measure of relationship functioning rather than family functioning, and reported that it was unrelated to depressive symptoms [9]. More research is needed to investigate the association between family functioning, relationship functioning, and depressive symptoms for mothers of preterm infants to inform the development of interventions to strengthen family functioning during NICU hospitalization with the intention to decrease stress and depressive symptoms.

Low social support was a significant predictor of depressive symptoms in the present study. This finding is consistent with meta-analyses that have reported low social support as strongly associated with depressive symptoms in mothers of term infants [1,3,10,25,26]. Independent of preterm or term infant status, Logsdon et al. (2001) also reported that low social support was strongly associated with depressive symptoms [53]. By contrast, two studies conducted in mothers of preterm infants reported no association between social support and depressive symptoms [8,28]. The measurement of social support in the aforementioned studies [8,28] differed from our study. Davis and colleagues [8] addressed nursing support and family support, and reported that low support from nurses, rather than family support, predicted depressive symptoms. Lefkowitz and colleagues [28] addressed perceived social support using a non-validated checklist. The use of a reliable measure for social support, and a larger sample size in part may account for our finding in mothers of preterm infants. Another explanation may be our heterogeneous sample with a large proportion of immigrant mothers who reported significantly less social support than Canadian born mothers. Immigrant mothers may be physically and culturally separated from their extended family support systems [14]. From a clinical perspective, supports (e.g., social and professional) beyond those currently available need to be considered in order to buffer the risk of depressive symptoms progressing to depression. In all three NICU research sites, mothers had open access to their infants, the support of health professionals (e.g., social workers, nurses) and parent support groups, yet compared to Canadian born mothers, lower support was reported by immigrant mothers. Further research is needed to address the unique needs for social support and professional support for mothers of preterm infants, and to conduct an analyses of immigrant and Canadian born mothers separately to investigate what support interventions best match the needs of immigrant and Canadian born mothers with preterm infants.

Similar to Miles and colleagues [9], in this study single parent status predicted depressive symptoms. In one meta-analysis, single parent status was a predictor of depression in mothers of term infants [26]. However, O’Hara [1] described marital status as less strongly related to depressive symptoms than psychosocial factors or history of depression. Overall, single mothers may have increased depressive symptoms related to low social support (i.e., without partner support) and lower income and employment. The additive effect of low resources, low support, and an ill preterm infant hospitalized in NICU may account for this factor being a significant predictor of depressive symptoms for single parents. The percentage of mothers who were single parents was similar between immigrant (9.4%) and Canadian born (7.1%) mothers. It cannot be assumed that the mechanisms underlying depressive symptoms are the same for single immigrant and single Canadian born mothers. Overall, immigrant mothers reported significantly lower social support and family functioning than Canadian born mothers. The cumulative effect of being single, an immigrant and experiencing the psychosocial strain related to preterm birth merits further investigation.

A strength of this study was the measurement of the infant’s severity of illness with a validated instrument (SNAP-II). The SNAP-II is limited in that severity of illness is measured at NICU admission and although correlated with therapeutic intensity and length of stay [49], it does not address illness severity throughout the infant’s NICU hospitalization. No other researchers have used a validated severity of illness measure. Rather, Miles and colleagues [9] measured cumulative risk for neurological insults during hospitalization. The SNAP-II and cumulative risk both failed to predict maternal depressive symptoms similar with earlier studies where infant demographic characteristics were used as a proxy for illness severity [8,9,28,30,54]. Maternal perception of her infant’s health (e.g., psychosocial factors) rather than objective indicators of illness severity may make a greater contribution to understanding the risk for depressive symptoms [9].

Factors identified as predictors of depressive symptoms in mothers of preterm infants during the NICU hospitalization period that were not addressed in this study included prior history of depression and/or anxiety [28,30] and knowledge of infant development [51]. Consistent with meta-analyses from studies with mother of term infants [1,25,26], maternal education level and first born children were non-predictive of depressive symptoms in this study. Income adequacy was not associated with depressive symptoms in this study different from meta-analyses that report low income as associated, although less strongly, with depressive symptoms in mothers of term infants [1,25,26]. The fact that maternal educational level and income adequacy were not significant predictors in our study may be related to the fact that the sample included primarily mothers with higher education and income [31].

Clinical implications are derived from the study’s findings and relate to strategies targeted at reducing the risk for depressive symptoms in mothers of preterm infants. For practice, our findings highlight the need to screen all mothers, especially those at greatest risk for depressive symptoms (e.g., single parent status, high stress, poorer family functioning, and less social support) while in the NICU setting. Interventions should focus on reducing stress and supporting coping strategies for all mothers. The stress related to the preterm infant and concurrent stressors may differ greatly across mothers. Single mothers and immigrant mothers may need additional and different types of support than married or Canadian born mothers. Social support interventions are required in addition to those supports currently offered in the NICU. Nurses have a supportive influence as evidenced by Davis et al [8]. Nurses have a continuous presence in caring for infants and their mothers and have the greatest opportunities to identify needs and seek out additional supports. In a small Canadian study, Preyde and Ardal [55] reported that mothers who voluntarily participated in a NICU-based peer support intervention had significantly less stress and anxiety, and greater perceived social support than mothers of preterm infants who did not receive the intervention. While this study cannot be generalized due to volunteer bias and a small sample, the effectiveness of a peer support intervention for mothers of preterm infants merits further investigation in a randomized controlled trial. Longitudinally, maternal depressive symptoms with low social support were associated with lower cognitive functions in preterm infants [56,57].

### Limitations

This study has a number of limitations. First, depression was measured using a self-report measure (CES-D) that was originally validated in the general adult population and used to screen for postpartum depression. As such, measurement error may occur because some questions on this general depression measure overlap with typical postpartum adjustment symptoms (e.g., sleeping difficulty). As well, mothers may screen positive for depressive symptoms but may not meet more rigorous depression specific criteria for a diagnosis of depression. Second, one of the known predictors of postnatal depression, history of depression [1] was not addressed in this study. Third, immigrant status was based on the mother’s birth country being other than Canada. This approach did not take into consideration the number of years in Canada which is an important consideration once residence is longer term (greater than 10 years) [19]. Lastly, the mothers in this study were primarily married, highly educated and over half were classified as having upper middle or high-income adequacy [31]. The study findings can be generalized only to mothers and preterm infant populations with similar characteristics, and the benefit of universal access to health care coverage as in Canada.

## Conclusions

In summary, these data provide the first view that immigrant mothers of preterm infants are at increased risk for depressive symptoms compared to Canadian born mothers. Further, these findings provide additional support that four factors (single parent status, high perceived stress, poorer family functioning and less social support) are important and significant predictors of depressive symptoms for immigrant and Canadian born mothers of preterm infants. For mothers of preterm infants hospitalized in NICU, and particularly for single mothers, interventions to reduce stress and increase family functioning and social support are required to reduce the development of depressive symptoms. Ultimately, by providing additional interventions the consequences of maternal depressive symptoms that are associated with negative child outcomes may also be reduced.

## List of abbreviations used

BW: birth weight; CES-D: Center for Epidemiologic Studies Depression Scale; FAD-GF: Family Assessment Device – General Functioning Scale; GA: gestational age; NICU: Neonatal Intensive Care Unit; PSS:NICU: Parental Stressor Scale: Neonatal Intensive Care Unit; SNAP-II: Score for Neonatal Acute Physiology-II; SSI: Social Support Index; VIA: Vancouver Index of Acculturation - Mainstream and Heritage Scales.

## Competing interests

The authors declare that they have no competing interests.

## Authors’ contributions

MB and KMB developed the design for the research with input from BT. MB and BT analyzed the data. MB, KMB, and BT interpreted the data. All authors read and approved the final version.

## Declarations

This article has been published as part of *BMC Pregnancy and Childbirth* Volume 13 Supplement 1, 2013: Preterm Birth: Interdisciplinary Research from the Preterm Birth and Healthy Outcomes Team (PreHOT). The full contents of the supplement are available online at http://www.biomedcentral.com/bmcpregnancychildbirth/supplements/13/S1.

All of the publication fees will be funded by the Preterm Birth and Healthy Outcomes Team Interdisciplinary Team Grant (#200700595) from Alberta Innovates - Health Solutions, formerly the Alberta Heritage Foundation for Medical Research.

## References

[B1] O'Hara MW Postpartum depression: what we know J Clin Psychol 2009 65 12 1258 1269 10.1002/jclp.20644 19827112

[B2] Gavin NI Gaynes BN Lohr KN Meltzer-Brody S Gartlehner G Swinson T Perinatal depression: a systematic review of prevalence and incidence Obstet Gynecol 2005 106 5 Pt 1 1071 1083 16260528 10.1097/01.AOG.0000183597.31630.db

[B3] Vigod SN Villegas L Dennis CL Ross LE Prevalence and risk factors for postpartum depression among women with preterm and low-birth-weight infants: a systematic review BJOG 2010 117 5 540 550 10.1111/j.1471-0528.2009.02493.x 20121831

[B4] Younger JB Kendell MJ Pickler RH Mastery of stress in mothers of preterm infants J Soc Pediatr Nurses 1997 2 1 29 35 10.1111/j.1744-6155.1997.tb00197.x 9051637

[B5] Pinelli J Saigal S Wu Y-W Cunningham C DiCenso A Steele S Austin P Turner S Patterns of change in family functioning, resources, coping and parental depression in mothers and fathers of sick newborns in the first year of life J Neonat Nurs 2008 14 5 156 165 10.1016/j.jnn.2008.03.015

[B6] Mew AM Holditch-Davis D Belyea M Miles MS Fishel A Correlates of depressive symptoms in mothers of preterm infants Neonat Netw 2003 22 5 51 60 10.1891/0730-0832.22.5.51 14598980

[B7] Cho J Holditch-Davis D Miles MS Effects of maternal depressive symptoms and infant gender on the interactions between mothers and their medically at-risk infants J Obstet Gynecol Neonatal Nurs 2008 37 1 58 70 10.1111/j.1552-6909.2007.00206.x PMC2718685 18226158

[B8] Davis L Edwards H Mohay H Wollin J The impact of very premature birth on the psychological health of mothers Early Hum Dev 2003 73 1-2 61 70 10.1016/S0378-3782(03)00073-2 12932894

[B9] Miles MS Holditch-Davis DR Schwartz TA Scher M Depressive Symptoms in Mothers of Prematurely Born Infants J Dev Behav Pediatr 2007 28 1 36 44 10.1097/01.DBP.0000257517.52459.7a 17353730

[B10] O'Hara MW Swain AM Rates and risk of postpartum depression - a meta-analysis Int Rev Psychiatry 1996 8 37 38 54

[B11] Singer LT Salvator A Guo S Collin M Lilien L Baley J Maternal psychological distress and parenting stress after the birth of a very low-birth-weight infant JAMA 1999 281 9 799 805 10.1001/jama.281.9.799 10071000 PMC10189739

[B12] Miles MS Gillespie JV Holditch-Davis D Physical and mental health in African American mothers with HIV J Assoc Nurses in AIDS Care 2001 12 4 42 50 10.1016/S1055-3290(06)60215-X 11486719

[B13] McGrath MM Sullivan MC Lester BM Oh W Longitudinal neurologic follow-up in neonatal intensive care unit survivors with various neonatal morbidities Pediatrics 2000 106 6 1397 1405 10.1542/peds.106.6.1397 11099595

[B14] Fung K Dennis CL Postpartum depression among immigrant women Curr Opin Psychiatry 2010 23 4 342 348 10.1097/YCO.0b013e32833ad721 20495458

[B15] Chiu T Tran K Maheux H Immigration in Canada: a portrait of foreign-born population, 2006 Census Ottawa: Statistics Canada 2007

[B16] Urquia ML Frank JW Moineddin R Glazier RH Does time since immigration modify neighborhood deprivation gradients in preterm birth? A multilevel analysis J Urban Health 2011 88 5 959 976 10.1007/s11524-011-9569-2 21503816 PMC3191215

[B17] Auger N Luo Z-C Platt R Daniel M Do mother’s education and foreign born status interact to influence birth outcomes? Clarifying the epidemiological paradox and the health migrant effect J Epidemiol Community Health 2008 62 402 409 10.1136/jech.2007.064535 18413452

[B18] Zelkowitz P Saucier JF Wang T Katofsky L Valenzuela M Westreich R Stability and change in depressive symptoms from pregnancy to two months postpartum in childbearing immigrant women Arch Womens Ment Health 2008 11 1 1 11 10.1007/s00737-008-0219-y 18270652

[B19] Miszkurka M Goulet L Zunzunegui MV Contributions of immigration to depressive symptoms among pregnant women in Canada Can J Public Health 2010 101 5 358 364 21214048 10.1007/BF03404853 PMC6973600

[B20] Sword W Watt S Krueger P Postpartum health, service needs, and access to care experiences of immigrant and Canadian-born women J Obstet Gynecol Neonatal Nurs 2006 35 6 717 727 10.1111/j.1552-6909.2006.00092.x 17105636

[B21] Mechakra-Tahiri S Zunzunegui MV Seguin L Self-rated health and postnatal depressive symptoms among immigrant mothers in Quebec Women Health 2007 45 4 1 17 10.1300/J013v45n04_01 18032165

[B22] Stewart DE Gagnon A Saucier JF Wahoush O Dougherty G Postpartum depression symptoms in newcomers Can J Psychiatry 2008 53 2 121 124 18357931 10.1177/070674370805300208

[B23] Ryder AG Alden LA Paulhus DL Is acculturaion unidimensional or bidimensional: A head-to-toe comparison in the prediction of personality, self-identity, and adjustment J Pers Soc Psychol 2000 79 49 65 10909877 10.1037//0022-3514.79.1.49

[B24] American Psychological Association Guidelines on Multicultural Education, Training, Research, Practice, and Organizational Change for Psychologists Am Psychol 2003 58 5 377 402 12971086 10.1037/0003-066x.58.5.377

[B25] Robertson E Grace S Wallington T Stewart DE Antenatal risk factors for postpartum depression: a synthesis of recent literature Gen Hosp Psychiatry 2004 26 4 289 295 10.1016/j.genhosppsych.2004.02.006 15234824

[B26] Beck CT Predictors of postpartum depression: an update Nurs Res 2001 50 5 275 285 10.1097/00006199-200109000-00004 11570712

[B27] Carter JD Mulder RT Bartram AF Darlow BA Infants in a neonatal intensive care unit: parental response Arch Dis Childhood Fetal Neonatal Ed 2005 90 2 F109 113 10.1136/adc.2003.031641 15724032 PMC1721867

[B28] Lefkowitz DS Baxt C Evans JR Prevalence and correlates of posttraumatic stress and postpartum depression in parents of infants in the Neonatal Intensive Care Unit (NICU) J Clin Psychol Med Settings 2010 17 3 230 237 10.1007/s10880-010-9202-7 20632076

[B29] Gennaro S Postpartal anxiety and depression in mothers of term and preterm infants Nurs Res 1988 37 2 82 85 3347524

[B30] Korja R Savonlahti E Ahlqvist-Bjorkroth S Stolt S Haataja L Lapinleimu H Piha J Lehtonen L Maternal depression is associated with mother-infant interaction in preterm infants Acta Paediatr 2008 97 6 724 730 10.1111/j.1651-2227.2008.00733.x 18373715

[B31] Ballantyne M Stevens B Guttmann A Willan AR Rosenbaum P Transition to neonatal follow-up programs: is attendance a problem? J Perinat Neonatal Nurs 2012 26 1 90 98 22293647 10.1097/JPN.0b013e31823f900b

[B32] Ontario Ministry of Finance, Commission on the reform of Ontario public services. Recent trends in immigration. 2012 http://www.fin.gov.on.ca/en/reformcommission/chapters/ch10.html

[B33] Radloff L The CES-D Scale: a self-report symptom scale to detect depression in a community sample App Psych Meas 1977 1 385 401 10.1177/014662167700100306

[B34] Campbell SB Cohn JF Prevalence and correlates of postpartum depression in first-time mothers J Abnorm Psychol 1991 100 4 594 599 1757673 10.1037//0021-843x.100.4.594

[B35] Holditch-Davis D Schwartz T Black B Scher M Correlates of mother-premature infant interactions Res Nurs Health 2007 30 3 333 346 10.1002/nur.20190 17514707

[B36] Mandl KD Tronick EZ Brennan TA Alpert HR Homer CJ Infant health care use and maternal depression Arch Pediatr Adolesc Med 1999 153 8 808 813 10437752 10.1001/archpedi.153.8.808

[B37] Miles MS Holditch-Davis D Burchinal P Nelson D Distress and growth outcomes in mothers of medically fragile infants Nurs Res 1999 48 3 129 140 10.1097/00006199-199905000-00003 10337844

[B38] Pridham K Lin CY Brown R Mothers' evaluation of their caregiving for premature and full-term infants through the first year: contributing factors Res Nurs Health 2001 24 3 157 169 10.1002/nur.1019 11526615

[B39] Spear ML Leef K Epps S Locke R Spear ML Leef K Epps S Locke R Family reactions during infants' hospitalization in the neonatal intensive care unit Am J Perinatol 2002 19 4 205 213 10.1055/s-2002-28484 12012282

[B40] Myers JK Weissman MM Use of a self-report symptom scale to detect depression in a community sample Am J Psychiatry 1980 137 9 1081 1084 7425160 10.1176/ajp.137.9.1081

[B41] Roberts RE Vernon SW The Center for Epidemiologic Studies Depression Scale: its use in a community sample A J Psychiatry 1983 140 1 41 46 10.1176/ajp.140.1.41 6847983

[B42] Miles MS Funk SG Carlson J Parental Stressor Scale: neonatal intensive care unit Nurs Res 1993 42 3 148 152 8506163

[B43] Epstein N Baldwin L Bishop D The McMaster family assessment device J Mar Fam Ther 1983 9 171 180 10.1111/j.1752-0606.1983.tb01497.x

[B44] Byles J Byrne C Boyle MH Offord DR Ontario Child Health Study: reliability and validity of the general functioning subscale of the McMaster Family Assessment Device Fam Process 1988 27 1 97 104 10.1111/j.1545-5300.1988.00097.x 3360100

[B45] Pinelli J Effects of family coping and resources on family adjustment and parental stress in the acute phase of the NICU experience Neonatal Netw 2000 19 6 27 37 10.1891/0730-0832.19.6.27 11949118

[B46] Doucette J Pinelli J The effects of family resources, coping, and strains on family adjustment 18 to 24 months after the NICU experience Adv Neonatal Care 2004 4 2 92 104 10.1016/j.adnc.2004.01.005 15138992

[B47] McCubbin HP J Glynn T Social Support Index (SSI) 1982 Madison, WI: Family Stress, coping and health project, school of human ecology, University of Wisconsin

[B48] Richardson DK Gray JE McCormick MC Workman K Goldmann DA Score for Neonatal Acute Physiology: a physiologic severity index for neonatal intensive care Pediatrics 1993 91 3 617 623 8441569

[B49] Richardson DK Tarnow-Mordi WO Escobar GJ Neonatal risk scoring systems. Can they predict mortality and morbidity? Clin Perinatol 1998 25 3 591 611 9779336

[B50] Richardson DK Corcoran JD Escobar GJ Lee SK SNAP-II and SNAPPE-II: Simplified newborn illness severity and mortality risk scores J Pediatr 2001 138 92 100 10.1067/mpd.2001.109608 11148519

[B51] Veddovi M Kenny DT Gibson F Bowen J Starte D The relationship between depressive symptoms following premature birth, mothers' coping style, and knowledge of infant development J Reprod Infant Psychol 2001 19 4 313 323

[B52] Dennis CL Ross LE Depressive symptomatology in the immediate postnatal period: identifying maternal characteristics related to true- and false-positive screening scores Can J Psychiatry 2006 51 5 265 273 16986815 10.1177/070674370605100501

[B53] Logsdon MC Usui W Psychosocial predictors of postpartum depression in diverse groups of women West J Nurs Res 2001 23 6 563 574 11569330 10.1177/019394590102300603

[B54] Shaw RJ Bernard RS Deblois T Ikuta LM Ginzburg K Koopman C The relationship between acute stress disorder and posttraumatic stress disorder in the neonatal intensive care unit Psychosomatics 2009 50 2 131 137 10.1176/appi.psy.50.2.131 19377021

[B55] Preyde M Ardal F Effectiveness of a parent "buddy" program for mothers of very preterm infants in a neonatal intensive care unit CMAJ 2003 168 8 969 973 12695379 PMC152679

[B56] McManus BM Poehlmann J Maternal depression and perceived social support as predictors of cognitive function trajectories during the first 3 years of life for preterm infants in Wisconsin Child Care Health Dev 2012 38 3 425 434 10.1111/j.1365-2214.2011.01253.x 21651608 PMC3191240

[B57] Poehlmann J Schwichtenberg AJ Bolt D Dilworth-Bart J Predictors of depressive symptom trajectories in mothers of preterm or low birth weight infants J Fam Psychol 2009 23 5 690 704 19803605 10.1037/a0016117 PMC2791691

